# Supplementary material for: Serologic Investigation on Tick-Borne Encephalitis Virus, Kemerovo Virus and Tribeč Virus Infections in Wild Birds
Source: Microorganisms. 2022 Dec 2;10(12):2397. doi: 10.3390/microorganisms10122397 (PMC9784810; doi:10.3390/microorganisms10122397)
Supplement: Supplementary file 1 [file microorganisms-10-02397-s001.zip › microorganisms-2009917-supplementary.pdf]

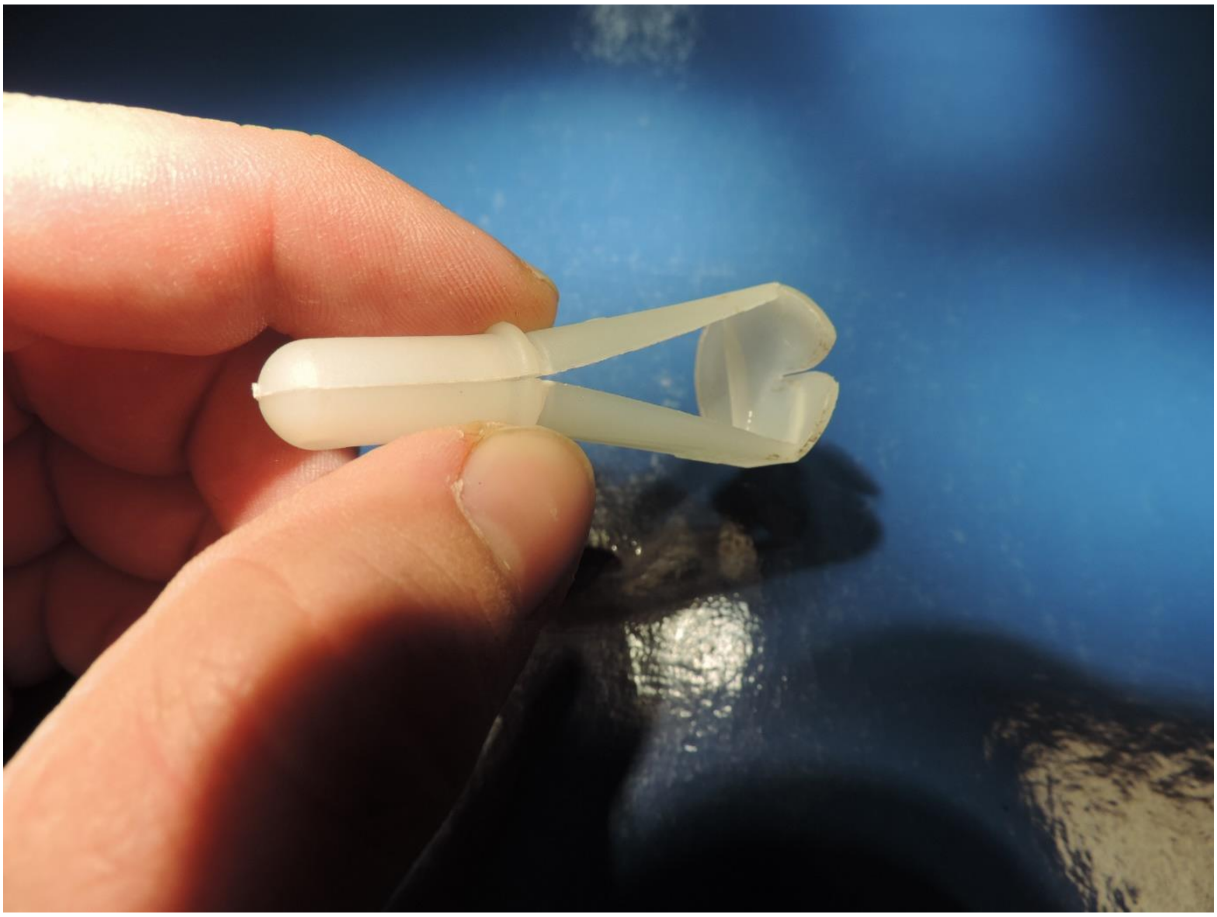

Figure S1 Tick removal spoon (Dr. Kapiller®, Budapest, Hungary)

Table S1. Thermal profiles of PCR used for the GIV serogroup orbivirus RNA and *Flavivirus* RNA detection

GIV serogroup orbivirus PCR		
Initial denaturation	1×	95 °C, 1 min
Denaturation	40×	95 °C, 45 sec
Annealing		55 °C, 30 sec
Extension		72 °C, 45 sec
Final extension	1×	72 °C, 10 min
Flavivirus generic PCR<br>1st PCR		
Initial denaturation	1×	95 °C, 1 min
Denaturation	35×	95 °C, 30 sec
Annealing		50 °C, 45 sec
Extension		72 °C, 45 sec
Final extension	1×	72 °C, 5 min
2nd PCR		
Initial denaturation	1×	95 °C, 1 min
Denaturation	40×	95 °C, 30 sec
Annealing		50 °C, 45 sec
Extension		72 °C, 45 sec
Final extension	1×	72 °C, 5 min

Table S2. The list of captured bird species and proportion of seropositive tick-infested and non-infested individuals in the screening and their migratory status

Bird species	No. of caught birds/ No. of tick-infested individuals	Migratory status	No. of seropositive / no. of tested						No. of co-infection (cross-reaction) per individual		
			Non-infested birds			Tick-infested birds			TBEV+ TRBV	TBEV+ KEMV	TRBV+ KEMV
			TBEV	TRBV	KEMV	TBEV	TRBV	KEMV			
Acrocephalus arundinaceus/ Great reed warbler	2/0	L	0/2	0/1	0/2	-	-	-	-	-	-
Acrocephalus palustris/ Marsh warbler	10/0	L	0/10	2/6	1/7	-	-	-	-	-	-
Acrocephalus scirpaceus/ Reed warbler	1/0	L	0/1	0/1	0/1	-	-	-	-	-	-
Alcedo atthis/ Common kingfisher	1/0	S	0/1	0/1	NT	-	-	-	-	-	-
Buteo buteo/ Common buzzard	1/0	S	0/1	NT	0/1	-	-	-	-	-	-
Carduelis carduelis/ European goldfinch	2/0	S	0/2	0/1	0/1	-	-	-	-	-	-
Coccothraustes coccothraustes/ Hawfinch	14/3	S	0/11	2/8	0/10	0/3	3/3	1/3	-	-	$0^{ni}/1^{i}$
Cyanistes caeruleus/ Eurasian blue tit	7/0	S	0/7	0/2	0/4	-	-	-	-	-	-
Emberiza citrinella/ Yellowhammer	4/0	S	0/4	0/3	0/3	-	-	-	-	-	-
Emberiza schoeniclus/ Common reed bunting	1/0	S	0/1	NT	0/1	-	-	-	-	-	-
Erithacus rubecula/ European robin	93/23	S	5/69	6/36	2/57	1/22	4/22	1/22	$1^{ni}/1^{i}$	-	$2^{ni}/1^{i}$
Ficedula albicollis/ Collared flycatcher	1/0	L	0/1	NT	0/1	-	-	-	-	-	-
Fringilla coelebs/ Common chaffinch	11/3	S	0/8	1/3	2/6	0/3	1/3	0/3	-	-	$1^{ni}/0^{i}$
Fringilla montifringilla/ Brambling	1/0	S	0/1	NT	0/1	-	-	-	-	-	-

Table S2. *Continued*

Bird species	No. of caught birds/ No. of tick-infested individuals	Migratory status	No. of seropositive / no. of tested						No. of co-infection (cross-reaction) per individual		
			Non-infested birds			Tick-infested birds			TBEV+ TRBV	TBEV+ KEMV	TRBV+ KEMV
			TBEV	TRBV	KEMV	TBEV	TRBV	KEMV			
Garrulus glandarius/ Eurasian jay	3/0	S	0/3	1/3	2/3	-	-	-	-	-	$1^{ni}/0^{i}$
Chloris chloris/ European greenfinch	1/0	S	0/1	NT	0/1	-	-	-	-	-	-
Lanius collurio/ Red-backed shrike	2/0	L	0/2	0/2	0/2	-	-	-	-	-	-
Luscinia megarhynchos/ Common nightingale	10/2	L	1/8	0/5	0/8	0/2	0/2	0/2	-	-	-
Parus major/ Great tit	72/18	S	0/54	8/36	2/47	0/14	1/14	1/14	-	-	$2^{ni}/0^{i}$
Passer montanus/ Eurasian tree sparrow	1/0	S	0/1	0/1	NT	-	-	-	-	-	-
Phoenicurus ochruros/ Black redstart	1/0	S	0/1	0/1	0/1	-	-	-	-	-	-
Phyllocopus collybita/ Common chiffchaff	1/0	S/L	0/1	0/1	0/1	-	-	-	-	-	-
Poecile palustris/ Marsh tit	5/0	N	0/5	NT	0/3	-	-	-	-	-	-
Prunella modularis/ Dunnock	5/2	S	0/3	0/2	0/3	0/1	1/1	0/1	-	-	-
Pyrrhula pyrrhula/ Eurasian bullfinch	1/0	S	0/1	0/1	NT	-	-	-	-	-	-
Sitta europaea/ Wood nuthatch	2/0	N	0/2	0/2	0/2	-	-	-	-	-	-
Sylvia atricapilla/ Eurasian blackcap	93/3	S/L	24/90	4/58	7/69	0/3	0/3	0/3	$1^{ni}/0^{i}$	$1^{ni}/0^{i}$	-
Sylvia borin/ Garden warbler	1/0	L	0/1	NT	NT	-	-	-	-	-	-
Sylvia curruca/ Lesser whitethroat	1/0	L	0/1	NT	NT	-	-	-	-	-	-
Turdus iliacus/ Redwing	1/0	S	0/1	1/1	0/1	-	-	-	-	-	-

Table S2. *Continued*

Bird species	No. of caught birds/ No. of tick-infested individuals	Migratory status	No. of seropositive / no. of tested						No. of co-infection (cross-reaction) per individual		
			Non-infested birds			Tick-infested birds			TBEV+ TRBV	TBEV+ KEMV	TRBV+ KEMV
			TBEV	TRBV	KEMV	TBEV	TRBV	KEMV			
Turdus merula/ Common blackbird	31/27	S	2/4	1/4	0/4	4/21	10/21	5/21	$0^{ni}/1^{i}$	-	$0^{ni}/4^{i}$
Turdus philomelos/ Song thrush	13/7	S	0/4	0/3	0/4	0/5	4/5	0/5	-	-	-

Legend: no. – number; - – negative; NT – not tested, ^{ni} *– non-infested birds;* ^{i} *– infested birds; L – species strictly long-distance migrant, S – species strictly short-distance migrant, S/L – species in which some individuals are short-distance migrants whereas others are long-distance migrants, N – non-migratory species*
